# The roles of personal interview and cognitive abilities at admission to medical school in predicting performance of medical students in their internal medicine sub-internship

**DOI:** 10.1186/s12909-022-03614-1

**Published:** 2022-07-13

**Authors:** Idit F. Liberty, Lena Novack, Reli Hershkovitz, Amos Katz

**Affiliations:** 1 Diabetes Clinic, Soroka University Medical Center, Beer-Sheva, Israel; 2 Faculty of Health Sciences, Ben-Gurion University of the Negev, Beer-Sheva, Israel; 3 Soroka Clinical Research Center, Soroka University Medical Center, Beer-Sheva, Israel; 4 Department of Obstetrics and Gynecology, Soroka University Medical Center, Beer-Sheva, Israel

**Keywords:** Admission to medical school, Students’ performance, Internship, Medical personnel evaluation

## Abstract

**Background:**

The medical school admission process is complicated, perhaps reflecting unresolved debates concerning the most important skills necessary to become an ideal physician. The Goldman Medical School at Ben-Gurion University in Israel is known for placing great emphasis on the personal attributes of candidates in addition to their academic excellence. To this end, 1-h consecutive interviews are embedded in the admission process. This study aims to determine whether there is an association between candidates’ personal interview ratings and the ratings assigned to these students at the conclusion of their 6^th^ year internal medicine sub-internship.

**Methods:**

Our study sample included 136 students who were admitted to the medical school in 2015, and who completed their 6^th^ year internal medicine sub-internship in 2019–2020. Our data were derived from the admissions information for each candidate and from structured interviews concerning medical competence and personal traits, which were completed by medical personnel who were in contact with these students during their clinical rounds.

**Results:**

Higher interview ratings of candidates during the admission process were associated with a higher probability that students would be evaluated as top-rated internists 6 years later (Odds Ratio (OR) = 9.4, *p*-value = 0.049), independent of gender (OR for male vs female = 0.2, *p*-value = 0.025) and age (OR = 1.3 per each year, *p*-value = 0.115). Although significant, the numeric difference in interview rating was relatively small (median 9.5 and 9.4 for top-rated and not top-rated internists, respectively).

**Conclusions:**

Our study shows that high personal interview ratings assigned to candidates as part of the medical school admission process are predictive of high performance ratings of students after they complete their 6^th^ year internal medicine sub-internships. These findings demonstrate the value and importance of using semi-structured personal interviews in the medical school admission process.

**Supplementary Information:**

The online version contains supplementary material available at 10.1186/s12909-022-03614-1.

## Background

A fundamental question for medical schools around the world is how to select candidates most likely to become excellent physicians. In the past, it was common to rely only on candidates’ cognitive abilities and previous academic success because cognitive skills and academic grades were thought to predict medical school and career success [1]. However, the association between academic performance and success beyond medical school is relatively weak [2]. Some studies [3, 4] provide clear evidence that candidates selected on the basis of high academic performance alone are much more likely to drop out of medical school than candidates selected through a more complex admission process. Moreover, relying only on tools which measure cognitive abilities introduces a significant socio-economic class bias [5, 6]. Therefore, in recent years, there has been increasing agreement that medical school admission processes aimed at selecting candidates with the highest probability of becoming excellent physicians should include evaluation of two principal domains, (1) basic intelligence and cognitive aptitude, and (2) personality, including candidates’ conscientiousness, extraversion, self-esteem, and sociability [7]. Various tools have been developed to examine these traits, including personal interviews, biographical questionnaires, situational judgment tests, multiple mini-interviews, and other methods. Processes vary among medical schools, among countries, and within Israel, reflecting the diversity of vision and mission of particular schools.

### The Ben Gurion University Goldman Medical School admissions process

**Fig. 1:**
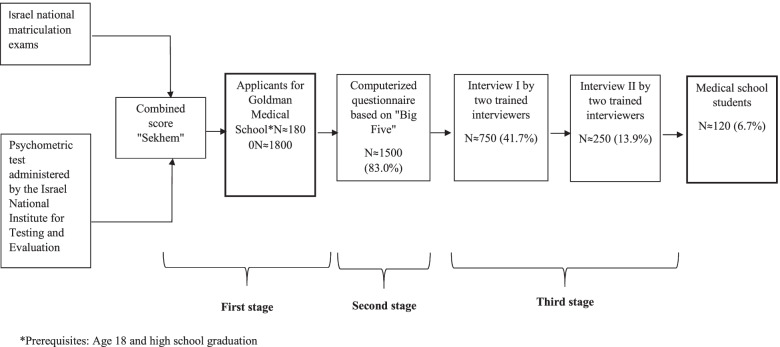
Goldman medical school admission process

Since its establishment in 1974, the six-year program at the Goldman Medical School of Ben-Gurion University (BGU) has placed great emphasis on the personal traits and skills of prospective students, and has therefore focused intensively on personality evaluations as a first domain priority once the basic (although very high) intelligence criterion has been met [8–11]. The admission process is open to all high school graduates and includes three stages (Fig. 1). The first stage involves presenting a sufficient “Sekhem” score. This score represents the weighted average of a candidate’s Israeli national matriculation examination scores and the score received on the psychometric test administered by the Israel National Institute for Testing and Evaluation. A sufficient Sekhem score is a prerequisite for applying for admission to medical schools in Israel. At BGU, the Sekhem average historically has been set lower relative to other medical schools in Israel, given BGU’s outlook that the highest Sekhem scores do not necessarily predict the best medical students and physicians [12]. Additionally, a slightly lower Sekhem requirement promotes greater socioeconomic diversity among candidates.

Candidates with sufficient Sekhem scores advance to the second stage of the admission process, which is comprised of a computerized questionnaire based on the “Big Five” [13], which evaluates different personality variables, including conscientiousness, extraversion, agreeableness, openness to experience, and emotional stability. This tool “widens the funnel” so all candidates with the necessary basic cognitive abilities are given an opportunity to be further considered based on strong personal traits and skills.

The candidates with the highest scores on the computerized questionnaire are invited for an interview by two trained interviewers in a session that may take up to one hour (Stage 3). One interviewer is a physician and the other is a community representative. Community representatives must have earned at least a bachelor’s degree Community representatives come from many disciplines including law, research, engineering, psychology, social welfare, education and other fields. All interviewers must be approved by the admission committee, and participate in workshops involving interview skills and protocol. Approximately 120 interviewers are involved in each intake.

Interviews focus on candidates’ pre-written structured resumes and interview questions that inquire into specific aspects of candidates’ qualifications, including thinking ability, emotional ability, social skills, social awareness and social involvement, as well as the ability to perform well under pressure.

With the goal of achieving maximal reliability and validity of interview ratings, interviewers are trained to adhere to the predetermined scope and content of the interview questions.

Additionally, the scores for each of the interviews are standardized separately as to each interviewer by calculating a t-score relative to the particular interviewer’s grading history. Thus, the t-score of the grade indicates the extent to which a candidate scored high or low relative to other evaluations of the same interviewer. The averaged t-scores for both interviewers represent the final interview score. The scores are back-transformed into grades 0–10 for final comparison.

Over the years, the admission process has varied in terms of the interview protocol. It has typically included 2 consecutive interviews (involving 4 interviewers), with the second interview being administered only to candidates who achieved the necessary threshold score in the first. The “2-interview” protocol has always been preferred over the “1-interview” structure, but has depended on the capacity of the admission committee to provide a sufficient number of interviewers from year to year.

Out of approximately 1,800 candidates, the 120 candidates with the highest overall score are admitted, which constitutes an acceptance rate of around 7%. Graduates of the Goldman Medical School have become known for their excellent clinical and interpersonal skills. Former BGU faculty members, Friedberg & Glick (1997) [14] analyzed evaluations made by department heads in hospitals throughout Israel comparing BGU graduates to their counterparts trained at other Israeli medical schools. Seventy-four percent (74%) of department heads thought that BGU graduates were better in physician–patient relations, and 49% felt they excelled in physician-team relations, as compared to those trained elsewhere. While these are favorable findings, the relationship between BGU’s emphasis on assessing human factors in the medical school admission process and actual student performance has never been evaluated quantitatively.

### This study

We examined the association between admission process ratings of candidates’ personal traits and skills as determined by personal interviews during the admissions process, and the students’ performance ratings after their completion of the 6^th^ year internal medicine rotation. The rotation is practically a six-week sub-internship designed to prepare students for their role as in-house internists. During the rotation, students are required to function as would a first-year resident under the supervision of senior medical personnel. Thus, students assume primary responsibility for patients that the students admit to the department. Students are evaluated as to their skills to assess patients accurately and efficiently, diagnose, provide immediate care, treat general medical conditions requiring hospitalization, and provide for an appropriate discharge and safe transition to community-based care. Students are assessed as to the problem-solving skills needed to facilitate future medical care/compliance in a patient population frequently without prior medical care, and to effectively communicate with patients, families, colleagues, consultants, primary care physicians, and allied health professionals. The skills evaluated in the 6th year internal medicine rotations have high fidelity with those needed in their careers. Therefore, the performance ratings in this rotation can be considered a proxy measure for future performance as physician.

## Methods

Our study sample included 136 students who were admitted to the medical school in 2015, and who completed their 6^th^ year internal medicine sub-internship in 2019–2020. Our data were derived from the admissions information for each candidate and from structured questionnaires concerning these students’ medical competence and personal traits, which were completed by medical personnel who were in contact with these students during their clinical rounds. We also considered 6^th^ year self-evaluations completed by these students concerning their medical studies and medical career aspirations. The study was approved by the institutional review board of the Faculty of Health Sciences at Ben Gurion University and conducted in accordance therewith.

### Medical personnel questionnaire

In order to obtain reliable data concerning students’ performance as physicians – the main study outcome – we approached all medical personnel who were in contact with students during their clinical rounds in 7 internal medicine departments at Soroka University Medical Center (SUMC) in Beer-Sheva, Israel and in 2 internal medicine departments at the Assuta Hospital in Ashdod, Israel. The maximum number of students in each department was 8. Both hospitals are academically affiliated with the BGU. The researchers (IL, LN and a research assistant) sought evaluations from the heads of the departments, senior residents in charge of students, and the departments’ head nurses. The median number of evaluators who were sought out in relation to each student was 3.

We contacted the medical personnel within 2 weeks of the students’ completion of their clinical round to ensure the evaluators’ memory on the students’ performance was relatively free of recall bias. For the same reason, the questionnaire for each student included the student’s passport photo. The questionnaire queried personnel concerning students’ fitness to become physicians and their personal traits and skills vis a vis the practice of medicine. These evaluations were completed independently by personnel in contrast to being administered in a group setting. (See the medical personnel questionnaire, Appendix 1, Supplementary materials). The particular student’s name was later removed from the questionnaire after being merged with the rest of the student’s information. We used the student’s national ID number to ensure accurate merger of all information. The structured format of the questions was designed to ensure sufficient reliability of evaluation ratings. The validity of these assessments was fortified by dichotomizing the scores as will be explained below.

The definition of “top-rated internist” was based on the two main questionnaire items: (1) Whether the personnel members wanted the particular student to work in their department in the future, and (2) the particular student’s sociometric positioning within the group of students who worked in the department. The student was defined as a “top-rated internist” if all evaluators of the student expressed a desire to work with the student in the future (graded as 6 out of 6) and/or the student was positioned as 1st by all of the evaluators as to sociometric rank.

We extracted information as to all grades received by the students during their 6 years of medical school study. Additionally, we administered a short questionnaire to the 6^th^ year students in order to elicit their views of their medical school experience and future career directions. (See the students’ questionnaire, Appendix 2, Supplementary materials.) The students’ questionnaire responses were identified by national ID number, and de-identified once combined into the analysis.

### Statistical analysis

All continuous variables, including age, final grade at admission (and its four composites, interview grade, matriculation examination grades, psychometric grade, and computerized personality test), as well as grades earned in courses during medical school, and ordinal scores for satisfaction with the choice of “medical doctor” as a career and interest in pursuing medical research, were described using mean ± standard deviation (SD), median, minimum and maximum. Age was compared among the study sub-groups using a t-test. For the comparisons of all other variables, we used a Wilcoxon signed-rank test, a non-parametric alternative, as we expected that the distribution of our results would depart from the Gaussian curve. Categorical variables, which included gender, family status, religiosity, service in the army, type of army unit, high school location, whether the student was pursuing an additional degree, and choice of medical specialization, were presented as percentages and compared among groups using a Chi-square test. The Fisher Exact test was used in cases with more than 25% of the cells, with an expected count of less than five, specifically in comparing the variables of army service and preferences of ER/ICU, orthopedics, ophthalmology, and dermatology. We used intraclass correlation (ICC) to assess reliability of different raters in relation to each student. We used a multivariable analysis to elaborate upon students’ evaluations of their roles as medical professionals (as a dependent variable) by age and gender (independent factors), using logistic and Poisson regression modeling. As a part of the sensitivity analysis, the model was repeated in a subset of students evaluated by at least 2 or 3 medical personnel members. We considered *p*-value ≤ 0.05 in a two-sided test to be statistically significant.

The study was in accordance with the relevant national/institutional guidelines.

## Results

We collected the data of 136 students overall. Of those, 115 students (88.5%) were admitted to the medical school in 2015 and the rest started their studies in earlier years but postponing their graduation due to their enrollment in a joint-degree program (MD-PhD, MD-MSc, etc.). The admission data for 3 students who were admitted prior to 2012 were unavailable to the researchers.

Data for 110 students were received from the medical personnel with respect to 6^th^ year students’ internal medicine sub-internship performance. Data for 26 students were unavailable owing to complicated logistics related to the onset of the COVID-19 pandemic half-way through the course of our study. Some medical personnel declined to provide sociometric evaluations to the researchers on ethical grounds. Therefore, student ranking data was unavailable as to 17 of the 110 students (15%). The researchers received 134 completed personal questionnaires from the students.

The teams of medical personnel (raters) who provided evaluations included between 1 and 7 people per team, with half of the students having been graded by 3 and more raters. The ICC for the questions ranged between 0.22–0.54, and was equal to 0.42 for the main question used in the analysis. With respect to 31 of 107 students (29.0%), there were unanimous departmental ratings of 6 out of 6 for the question of whether raters wanted the student to work in their department in the future. The results revealed that 6 of 93 students (6.5%) were ranked by medical personal as first in their internal medicine sub-intern group, and that 34 of 108 students (31.5%) met one of the two conditions above, and therefore were defined as “top-rated internists,” while the other students were defined as “not top-rated internists.” Descriptive tables are stratified by “top-rated internist” and “not top-rated internist” ratings (Table 1).

**Table 1:** Demographic characteristics by ratings of students as internists

Students’ characteristics	Top-rated internists (*N* = 34)	Not top-rated internists (*N* = 74)	*p*-value
Male gender, % (n/N)	22.6 (7/33)	35.3 (24/68)	0.150
Age at the 6^th^ year, years			
Mean ± SD (n)	30.2 ± 2.0 (25)	29.7 ± 2.0 (49)	0.360
Median	30.0	30.0	
Min; Max	26.0; 35.0	26.0; 33.0	
Family status, % (n/N)			
Married	48.2 (13/27)	49.0 (25/51)	0.942
Single	51.9 (14/27)	51.9 (26/51)	
Divorced	0.0 (0/27)	0.0 (0/51)	
Religiosity, % (n/N)			
Religious	18.5 (5/27)	20.0 (10/50)	0.456
Traditional	14.8 (4/27)	6.0 (3/50)	
Secular	66.7 (18/27)	74.0 (37/50)	
Served in IDF, % (n/N)	92.6 (25/27)	82.4 (42/51)	0.311
IDF unit, % (n/N)			
Combat	4.0 (1/25)	17.1 (1/41)	0.021
Elite forces	4.0 (1/25)	26.8 (11/41)	
Education corps	16.0 (4/25)	2.4 (1/41)	
Air force	8.0 (2/25)	4.9 (2/41)	
Intelligence forces	36.0 (9/25)	22.0 (9/41)	
Medical corps	0.0 (0/25)	12.2 (5/41)	
Navy	12.0 (3/25)	2.4 (1/41)	
Management	4.0 (1/25)	4.9 (2/41)	
Recruiting unit	4.0 (1/25)	0.0 (0/41)	
Combat support	12.0 (3/25)	2.4 (1/41)	
National service	0.0 (0/25)	2.4 (1/41)	
Radio	0.0 (0/25)	2.4 (1/41)	
High school geographic region, % (n/N)			
Jerusalem	11.5 (3/26)	26.5 (13/49)	0.293
Tel-Aviv—Center	42.3 (11/26)	36.7 (18/49)	
Haifa—North	34.6 (9/26)	18.4 (9/49)	
Southern Region	3.9 (1/26)	2.0 (1/49)	
Other	7.7 (2/26)	1.3 (8/49)	
Additional or joint degree during medical school, % (n/N)			
MPH	3.7 (1/27)	11.8 (6/51)	0.446
PhD	14.8 (4/27)	50.0 (4/51)	
Other degree	0.0 (0/27)	1.3 (1/51)	

On average, student age was 30.0 ± 2.0 years old, with 54.1% of them married, and 70.2% of them female. Over 70% of the students were secular and 88.8% served in the army. These characteristics did not vary between the top-rated internists and not top-rated internists, except with respect to top-rated internists whose army service involved the education corps, intelligence forces, or navy.

Students’ satisfaction with their choice of career or interest in pursuing medical research did not differ between top-rated internists and not top-rated internists (Table 2). However, a higher proportion of the latter group preferred the specialties of orthopedics, ophthalmology or dermatology.

**Table 2:** Students’ opinions about their medical studies and career direction by their ratings as internists

Students’ characteristics	Top-rated internists (*N* = 34)	Not top-rated internists (*N* = 74)	*p*-value
Satisfaction with MD choice as a future profession, scale 1–5			
Mean ± SD (n)	4.4 ± 0.6 (27)	4.5 ± 0.7 (50)	0.348
Median	4.0	5.0	
Min; Max	3.0; 5.0	2.0; 5.0	
Certainty concerning pursuit of research, scale 1–5			
Mean ± SD (m)	3.5 ± 0.9 (27)	3.2 ± 1.1 (50)	0.235
Median	4.0	3.0	
Min; Max	1.0; 5.0	1.0; 5.0	
Rated first, % (n/N)			
Internal Medicine / Pediatrics	54.2 (13/24)	45.5 (20/44)	0.492
OBGYN	17.4 (4/23)	26.8 (11/41)	0.392
Surgery	9.1 (2/22)	14.6 (6/41)	0.529
ER / ICU	5.3 (1/19)	11.9 (5/42)	0.655
Orthopedics	5.0 (1/20)	20.0 (8/40)	0.249
Ophthalmology / Dermatology	0 (0/21)	19.5 (8/41)	0.043

Comparison of students’ admission ratings with students’ 6^th^ year ratings as top-rated internists or not top-rated internists revealed diverse results vis a vis different aspects of the students’ admission ratings (Table 3). Specifically, the two groups varied only as to interview ratings, but not as to intelligence or computerized personality test ratings. Interview ratings were higher for the top-rated internists (with median 9.5 vs 9.4, *p*-value 0.013) as compared to all other admission process rating categories, including the Israel national matriculation exams, psychometric testing and the composite (Sekhem). Furthermore, the ratings from the computerized personality test were very nearly statistically even between top-rated internists and not top-rated internists (*p*-value = 0.922). The results showed that students who had ranked higher at the admission interview received higher 6^th^ year ratings for interaction with patients (*p*-value 0.044), accomplishing tasks (*p*-value 0.027), participating and being present (*p*-value 0.024), handling complex situations (*p*-value 0.020), and being reasonable (*p*-value 0.070) (Appendix 3, Supplementary materials).

**Table 3:** Students’ ratings at admission compared to their ratings after 6^th^ year internal medicine sub-internship

Students’ characteristics	Top-rated internists (*N* = 34)	Not top-rated internists (*N* = 73)	*p*-value
Final grade at admission^a^			
Mean ± SD (n)	9.5 ± 0.2 (34)	9.3 ± 0.4 (70)	0.020
Median	9.5	9.4	
Min; Max	8.8; 9.9	8.5; 10.0	
Interview grade averaged between the interviewers			
Mean ± SD (n)	9.5 ± 0.2 (34)	9.4 ± 0.3 (70)	0.013
Median	9.5	9.4	
Min; Max	8.9; 9.9	8.7; 9.9	
Israeli national matriculation exam			
Mean ± SD (n)	110.1 ± 2.7 (27)	110.1 ± 4.5 (63)	0.721
Median	110.4	110.1	
Min; Max	104.8; 115.6	89.4; 117.2	
Psychometric test			
Mean ± SD (n)	726.1 ± 24.1 (28)	720.5 ± 31.8 (64)	0.641
Median	726.5	724.5	
Min; Max	683.0; 776.0	518.0; 764.0	
Composite of matriculation exam & psychometric test (Sekhem)			
Mean ± SD (n)	772.2 ± 24.1 (34)	768.1 ± 34.0 (70)	0.966
Median	772.0	771.0	
Min; Max	736.0; 827.0	605.0; 815.0	
Computerized personality test			
Mean ± SD (n)	6.3 ± 0.7 (28)	6.2 ± 0.7 (64)	0.922
Median	6.3	6.2	
Min; Max	5.3; 8.0	5.0; 7.7	

^a^The score including interviews and “Sekhem,” i.e., composite of Israeli matriculation exam and psychometric test

Aiming to find medical school courses that were indicative of the top-rated internist or not top-rated internist assessment made by medical personnel, we analyzed course grades earned by the students during the 6-year medical curriculum. We chose the courses characterized by a coefficient of variance greater than 78 (on a scale 0–100), and therefore more likely to discriminate between top-rated internists and not top-rated internists. A simple comparison between the two groups did not yield a statistically significant result, but indicated a trend toward higher grades in two first-year courses, Physics and Body and Soul for students rated as top-rated internists in their 6^th^ year (Table 4).

**Table 4:** Students’ ratings at admission compared to their grades during medical school for selected courses^a^

Students’ characteristics	Top-rated internists (*N* = 34)	Not top-rated internists (*N* = 74)	*p*-value
Course: Physics			
Mean ± SD (n)	85.1 ± 8.5 (30)	82.5 ± 10.1 (62)	0.191
Median	86.0	79.5	
Min; Max	69.0; 100.0	66.0; 100.0	
Course: Organic Chemistry			
Mean ± SD (n)	84.4 ± 7.0 (28)	82.8 ± 9.0 (62)	0.539
Median	85.0	84.0	
Min; Max	69.0; 99.0	65.0; 100.0	
Course: Body and soul			
Mean ± SD (n)	86.1 ± 9.1 (34)	83.1 ± 9.1 (69)	0.135
Median	85.0	85.0	
Min; Max	65.0; 98.0	65.0; 98.0	
Course: Pharmacology			
Mean ± SD (n)	80.8 ± 8.8 (34)	80.1 ± 8.8 (70)	0.588
Median	82.0	78.0	
Min; Max	65.0; 98.0	67.0; 98.0	
Course: General Physiology & Electrophysiology			
Mean ± SD (n)	82.6 ± 7.8 (33)	82.7 ± 7.5 (69)	0.914
Median	83.0	82.0	
Min; Max	65.0; 96.0	63.0; 100.0	
Course: Biochemistry			
Mean ± SD (n)	88.3 ± 7.2 (33)	87.2 ± 8.5 (70)	0.583
Median	89.0	89.0	
Min; Max	74.0; 100.0	67.0; 100.0	
Course: Anatomy – limbs			
Mean ± SD (n)	81.3 ± 9.0 (31)	83.3 ± 8.1 (66)	0.380
Median	83.0	84.0	
Min; Max	61.0; 94.0	60.0; 97.0	
Course: Rheumatology and Clinical Immunology			
Mean ± SD (n)	79.5 ± 6.0 (32)	79.0 ± 7.4 (67)	0.553
Median	80.0	79.0	
Min; Max	65.0; 90.0	66.0; 97.0	
Course: Nephrological System			
Mean ± SD (n)	79.0 ± 6.7 (32)	78.8.3 ± 6.8 (67)	0.776
Median	79.0	77.0	
Min; Max	66.0; 93.0	65.0; 94.0	
Course: Neurosciences			
Mean ± SD (n)	81.5 ± 12.2 (29)	83.0 ± 7.9 (68)	0.978
Median	83.0	84.0	
Min; Max	32.0; 97.0	68.0; 100.0	
Course: Neuroanatomy			
Mean ± SD (n)	84.1 ± 5.9 (28)	83.6 ± 7.0 (65)	0.756
Median	85.0	84.0	
Min; Max	70.0; 92.0	67.0; 96.0	

^a^The courses were selected by their highest variability (Coeff of Var ≥ 8), and therefore with the highest potential to discriminate between “top-rated internist” and “not top-rated internist” ratings

Based on a multivariable analysis in a logistic regression, higher admission interview ratings were positively associated with higher chances of being evaluated as a top-rated internist 6 years later (OR = 9.4, *p*-value = 0.049), independent of students’ gender (OR for male vs female = 0.2, *p*-value = 0.025) and age (OR = 1.3 per each year, *p*-value = 0.115) (Table 5). Similar results were obtained in a sensitivity analysis by using a Poisson model, although with a borderline significance for the interview grade (*p*-value = 0.101 and relative risk = 4.0). A sensitivity analysis of the logistic model in a subset of students evaluated by at least 2 or 3 evaluators, indicated a similar direction in the association between the admission interview rating and the 6^th^ year top-rated internist rating. However, this association was of lower magnitude and not significant, specifically OR = 6.07 (*p*-value = 0.131) and OR = 2.09 (*p*-value = 0.628) for subsets with at least 2 and at least 3 evaluators, respectively.

**Table 5:** Factors at admission to the medical school associated with “top-rated internist” ratings: results of a multivariable logistic regression

Students’ characteristics	Odds Ratio	*p*-value
Interview grade (0–10)	9.4	0.049
Male gender	0.2	0.025
Age, years	1.3	0.115

## Discussion

In main thrust of this study was to examine the association between admission process candidate interview ratings and student performance ratings after completion of the 6th year internal medicine rotation. The main finding was that of all the components of the admissions process, the interview component had the highest correlation with students’ ratings at the conclusion of their 6^th^ year internal medicine rotation. Admissions process ratings derived from cognitive tests, the computerized personality test, as well as academic grades during the 6-year medical curriculum were not as highly correlated with students’ 6^th^ year internal medicine performance ratings.

Of note, the numeric difference in interview score was relatively small (median of 9.5 in top-rated vs. 9.4 in the not top-rated internists). This is mostly due to the low variability of scores in the study group limited to accepted, and therefore the best, candidates. The association between the interview and the students’ performance as internists, was established based on the higher rankings in the interview for the “top-rated internists” (reflected in a non-parametric test, Table 3) and later, the adjusted association based on the multivariable model (Table 5). The findings contradict most of the published research which report low reproducibility, and consequently, low validity of personal interviews conducted as part of the medical school admission process [15–17]. Our findings support the growing recognition of the importance of non-cognitive tests when considering candidates for admission to medical school. In a series of articles published in 2010, 2015 and 2020, Powis et al. [18–20] discuss the importance of selecting medical school candidates whose personal traits and skills suggest that they will provide high quality medical care to patients. These articles discuss various methods for examining non-cognitive parameters and the barriers to considering them at certain medical schools. The high cost of non-cognitive evaluations is cited as one such barrier. Another is some medical schools’ prioritization of “academic excellence” over personal traits and skills. Studies of the latter have been published in the field of organizational psychology, and mostly focus on admission to employment rather than to medical school [21]. Additional barriers arise from the unresolved debate as to whether a good medical student will necessarily be a good doctor[20, 22].

Our focus on the 6^th^ year internal medicine rotation ratings seemed to be the optimal measure at our disposal for examining student competence as future doctors. It is at this point in students’ medical training that senior physicians, residents and nurses get to know the students over a period of six weeks and have the opportunity to observe the students in terms of their medical knowledge, approach to patients, and teamwork with other health professionals.

Studies have shown that it is precisely in internal medicine that doctor-patient communication and the ability to conduct patient interviews is highly important and influences patients’ responses to treatment, patient satisfaction and treatment success [23]. Although good communication, empathy, and integrity are important qualities for all physicians, we would suggest that these attributes assume even greater importance in the field of internal medicine. Our medical school emphasis on the importance of personal factors such as these finds expression in the school’s carefully designed admission interview procedure. A study by Manuel et al. (2005) [24] reports a correlation between personality components and second year medical student clinical success in two parameters, (1) the Sixteen Personality Factor Questionnaire (16PF), and (2) the Clinical Skills Assessment II (CSA II). These findings reinforce our findings.

We found an association between medical personnel assessments of students and the students’ choices of internships later on. A higher percentage of students who were not top-rated internists chose to pursue dermatology, orthopedics and ophthalmology. Various studies have examined the relationship between different personality components and the choice of specialization. In one such study, Preece et al. (2016) [25], compared the personality components of surgery residents to medical students who expressed interest in pursuing surgery in the future. The study found that the personality components were similar between the two groups, as was the respective groups’ visual modality learning style. This research is consistent with our findings concerning not top-rated internists who preferred to work in surgical departments.

### Limitations

The obvious limitation of the study is not accounting for candidates who were not accepted to the medical school, and who might have been designated as top-rated internists in the 6^th^ year of medical study, thus representing a false negative fraction of the admission process. However, studying this group would have likely suffered from low participation given such factors as the candidates’ disappointment from not having been accepted by the medical school. Additionally, the group that was accepted comprised candidates with the highest interview ratings, which is what we wanted to compare with later ratings as 6^th^ year internists. Not surprisingly, this is reflected in the narrow range of ratings. In view of a relatively low variability among the accepted students, the difference of 0.1 in the admission interview ratings was statistically sufficient to differentiate between top-rated internists and not top-rated internists.

Another limitation is that we do not address whether 6^th^ year internal medicine rotation ratings predict success in the surgical or other non-internal medicine specialties.

As the study evaluated medical students at the end of medical school, future research is needed to determine how our findings may compare to findings as to practicing physicians.

## Conclusions

Our study shows that high personal interview ratings assigned to candidates as part of the medical school admission process are predictive of high performance ratings of medical students after they complete their 6^th^ year internal medicine sub-internship. These findings demonstrate the value and importance of using semi-structured personal interviews in the medical school admission process.

## Supplementary Information

**Additional file 1.**

## Data Availability

The datasets analyzed during the current study are not publicly available due the fact that this permission has not been granted by the ethics committee, however the de-identified information will be available from the corresponding author on reasonable request.
