# Supplementary material for: The roles of personal interview and cognitive abilities at admission to medical school in predicting performance of medical students in their internal medicine sub-internship
Source: BMC Med Educ. 2022 Jul 13;22:541. doi: 10.1186/s12909-022-03614-1 (PMC9281101; doi:10.1186/s12909-022-03614-1)
Supplement: Supplementary file 1 — Additional file 1. [file 12909_2022_3614_MOESM1_ESM.docx]

**Supplementary Material**

**Appendix 1:** Medical Staff evaluation form

Student's name_______________________ Student's ID _________________________

Medical personnel representative #_______

Medical personnel position in the department __________[senior physician/nurse/…]___

Department name __________________

*You have to express your opinion on the following statements on a scale 1-6 (using "√")*

| *Statement* | *1*  *Strongly disagree* | *2* | *3* | *4* | *5* | *6*  *Strongly agree* |
| --- | --- | --- | --- | --- | --- | --- |
| The student communicates with patients and their families in a way that shows sensitivity and compassion, while understanding the cultural background of the patient and his environment |  |  |  |  |  |  |
| The student cares about the patients and is willing to help medically and respond to their requests. |  |  |  |  |  |  |
| Maintains good relations with colleagues (medical, nursing and para-clinical staff) in a respectful and professional manner |  |  |  |  |  |  |
| The student performs his tasks properly: arrives and attends shifts as expected, supervises and is involved during the patients' hospitalization |  |  |  |  |  |  |
| Active partner in the department's activities (blood, X-ray meetings, literature sessions, etc.). His presence is very much felt in the activities of the department. |  |  |  |  |  |  |
| The student is able to analyze a complex situation, distinguishing between the primary and the secondary |  |  |  |  |  |  |
| The student demonstrates emotional resilience and draws conclusions while maintaining clinical discretion |  |  |  |  |  |  |
| The student demonstrates high motivation to succeed in his assignments and is willing to invest time and efforts for this purpose. |  |  |  |  |  |  |
| The student identifies himself with the system and is willing to invest efforts in advancing its goals. |  |  |  |  |  |  |

| *Statement* | *1*  *Strongly disagree* | *2* | *3* | *4* | *5* | *6*  *Strongly agree* |
| --- | --- | --- | --- | --- | --- | --- |
| The student is fit to be physician |  |  |  |  |  |  |
| You would like to see the student among my department doctors |  |  |  |  |  |  |

*Please rate all students you supervised during the rotation in you department, from highest to lowest. Grade 1 will be awarded to the student whose satisfaction is the highest.*

*Do not give a particular grade to more than one student.*

| **Rating** | **Student's name** |
| --- | --- |
| 1 |  |
| 2 |  |
| 3 |  |
| 4 |  |
| 5 |  |
| 6 |  |
| 7 |  |

**Appendix 2:** Students' evaluation form

Student's ID: _________________

In the questionnaire, only one answer must be chosen and / or specified in more details if necessary in the place provided

1. Gender male / female
2. Date of birth _____________
3. Marital status married/single/divorced
4. Religiousness status religious / traditional / secular
5. Town where you graduated from high school __________________
6. Did you serve in the army yes / no
7. If you served in the army, which unit did you serve in __________
8. Did you study additional degree during your studies at the medical school?
9. MPH
10. PhD
11. Another degree ___________
12. I did not study additional degree
13. Are you satisfied with your choice of being a physician?

| 1  Not at all | 2  Not so satisfied | 3  Moderately satisfied | 4  Quite satisfied | 5  Completely satisfied |
| --- | --- | --- | --- | --- |
|  |  |  |  |  |

1. Do you think that later in your career you will engage in research?

| 1  To a low degree | 2  Quite low | 3  Moderately agree | 4  Quite high | 5  To a high degree |
| --- | --- | --- | --- | --- |
|  |  |  |  |  |

1. What doctor would you like to be? The following options should be rated (numbered):

____Internal medicine / Pediatrics

____ OBGYN

____Surgery

____Intensive care / emergency room

____Dermatology / ophthalmology

____Other _______________

Every medical student encounters difficulties during their studies and clinical training.

Please select from the following list three areas in which you are experiencing difficulty, and rate them according to the intensity of the difficulty (1 - the most difficult). You can also specify domains that do not appear in the list:

_____ Academic requirements (memorization of study material, tests, etc.)

_____ Emotional overload as a result of meeting with patients

_____ Duration of training required for learning the profession

_____ Work under pressure and load conditions in the department

_____ The need to make decisions quickly, while incorporating extensive academic knowledge

_____ Work in a hierarchical framework with strict rules of conduct

_____ Dealing with frustration and anger of patients

_____ Maintaining good working relationships with department officials

_____Other: _________________________________

Appendix 3. Scores at admission by detailed evaluations by the medical personnel

| **Evaluation by the medical personnel** | **Composite score at admission** | | **Interview grade averaged between the interviewers** | | **National matriculation exam** | | **SAT** | | **Sekhem** | | **TIL** | |
| --- | --- | --- | --- | --- | --- | --- | --- | --- | --- | --- | --- | --- |
|  | Mean±SD (n)  Median | p-value | Mean±SD (n)  Median | p-value | Mean±SD (n)  Median | p-value | Mean±SD (n)  Median | p-value | Mean±SD (n)  Median | p-value | Mean±SD (n)  Median | p-value |
| **Sum of the 9 composites**:  below average  above average | 9.3±0.4 (37)  9.4  9.4±0.3 (66)  9.5 | 0.153 | 9.3±0.4 (37)  9.4  9.4±0.2 (66)  9.5 | 0.222 | 110.3±4.9 (33)  111.0  110.1±3.5 (56)  109.9 | 0.581 | 723.1±21.7 (34)  720.5  721.7±33.9 (57)  729.0 | 0.793 | 769.2±35.1 (34)  771.0  769.9±29.1 (57)  772.0 | 0.765 | 6.3±0.7 (34)  6.5  6.2±0.7 (57)  6.2 | 0.555 |
| **Interaction with patients (1)**:  below average  above average | 9.3±0.4 (38)  9.4  9.5±0.3 (65)  9.5 | 0.054 | 9.3±0.3 (38)  9.3  9.4±0.2 (65)  9.5 | 0.044 | 109.3±4.7 (33)  109.1  110.6±3.6 (56)  110.4 | 0.139 | 725.2±21.4 (34)  722.5  720.4±33.9 (57)  729.0 | 0.501 | 764.3±34.1 (34)  767.0  772.8±29.3 (57)  775.0 | 0.158 | 6.2±0.7 (34)  6.1  6.3±0.7 (57)  6.2 | 0.317 |
| **Cares about patients (2)**:  below average  above average | 9.3±0.4 (35)  9.4  9.4±0.3 (68)  9.5 | 0.232 | 9.3±0.3 (35)  9.4  9.4±0.2 (68)  9.5 | 0.382 | 109.9±3.9 (30)  109.2  110.2±4.2 (59)  110.4 | 0.430 | 726.0±24.6 (31)  724.0  720.2±34.5 (60)  729.0 | 0.519 | 768.5±24.3 (31)  770.0  770.2±34.6 (60)  773.5 | 0.527 | 6.3±0.7 (31)  6.2  6.2±0.7 (60)  6.2 | 0.446 |
| **Interaction w/staff (3)**:  below average  above average | 9.3±0.3 (33)  9.4  9.4±0.3 (70)  9.5 | 0.212 | 9.3±0.3 (33)  9.4  9.4±0.3 (70)  9.5 | 0.192 | 110.4±3.1 (29)  109.8  110.0±4.5 (60)  110.4 | 0.909 | 726.9±20.1 (30)  726.0  719.9±33.5 (61)  724.0 | 0.346 | 772.1±18.9 (30)  770.0  768.4±35.9 (61)  773.0 | 0.933 | 6.3±0.6 (30)  6.2  6.2±0.7 (61)  6.2 | 0.787 |
| **Accomplishes tasks (4)**:  below average  above average | 9.3±0.3 (40)  9.4  9.5±0.3 (63)  9.5 | 0.061 | 9.3±0.3 (40)  9.4  9.5±0.2 (63)  9.5 | 0.027 | 110.4±3.1 (35)  109.7  109.9±4.6 (54)  110.4 | 0.808 | 724.6±19.4 (36)  721.5  720.6±35.1 (55)  729.0 | 0.903 | 771.1±19.0 (36)  770.0  768.7±37.4 (55)  773.0 | 0.670 | 6.3±0.6 (36)  6.2  6.2±0.7 (55)  6.2 | 0.727 |
| **Participates and is present (5)**:  below average  above average | 9.3±0.3 (38)  9.4  9.5±0.3 (65)  9.5 | 0.015 | 9.3±0.3 (38)  9.4  9.5±0.2 (65)  9.5 | 0.024 | 110.2±5.0 (32)  110.7  110.1±3.5 (57)  110.1 | 0.700 | 722.0±21.9 (32)  716.0  722.3±33.7 (58)  730.0 | 0.488 | 768.2±35.1 (32)  770.0  770.4±29.2 (58)  772.5 | 0.997 | 6.3±0.7 (32)  6.2  6.2±0.7 (58)  6.2 | 0.763 |
| **Handles complex situations (6)**:  below average  above average | 9.3±0.3 (43)  9.4  9.5±0.3 (60)  9.5 | 0.034 | 9.3±0.3 (43)  9.4  9.5±0.2 (60)  9.5 | 0.020 | 109.7±4.8 (38)  109.7  110.4±3.5 (51)  110.4 | 0.460 | 724.4±20.9 (39)  724.0  720.6±35.2 (52)  729.0 | 0.751 | 768.6±33.5 (39)  770.0  770.5±29.9 (52)  772.5 | 0.873 | 6.3±0.7 (39)  6.2  6.2±0.7 (52)  6.2 | 0.449 |
| **Reasonable (7)**:  below average  above average | 9.3±0.4 (38)  9.4  9.5±0.3 (65)  9.5 | 0.080 | 9.3±0.3 (38)  9.4  9.4±0.2 (65)  9.5 | 0.070 | 109.6±4.7 (34)  109.5  110.7±3.6 (55)  110.4 | 0.332 | 724.1±20.5 (35)  725.0  721.0±35.2 (56)  729.0 | 0.851 | 766.4±34.1 (35)  770.0  771.7±29.5 (56)  774.0 | 0.424 | 6.3±0.6 (35)  6.2  6.2±0.7 (56)  6.2 | 0.648 |
| **Highly motivated (8)**:  below average  above average | 9.3±0.3 (39)  9.4  9.4±0.3 (64)  9.5 | 0.375 | 9.4±0.3 (39)  9.4  9.4±0.3 (64)  9.5 | 0.527 | 110.9±3.1 (34)  110.6  110.6±4.5 (55)  110.1 | 0.307 | 725.8±18.8 (35)  725.0  719.9±35.0 (56)  726.5 | 0.596 | 774.9±19.3 (35)  774.0  766.4±36.6 (56)  769.5 | 0.310 | 6.4±0.7 (35)  6.3  6.2±0.7 (56)  6.0 | 0.153 |
| **Identifies with the system (9)**:  below average (n=41)  above average (n=65) | 9.4±0.3 (38)  9.5  9.4±0.3 (65)  9.5 | 0.899 | 9.4±0.3 (38)  9.5  9.4±0.3 (65)  9.5 | 0.727 | 109.9±4.8 (34)  110.2  110.3±3.6 (55)  110.3 | 0.853 | 725.4±21.8 (35)  725.0  720.2±34.0 (56)  725.5 | 0.443 | 768.9±34.8 (35)  770.0  770.1±29.3 (56)  772.5 | 0.916 | 6.2±0.7 (35)  6.1  6.3±0.7 (56)  6.2 | 0.330 |
